# Glucose-6-phosphatase catalytic subunit 3 enhances host resistance to porcine epidemic diarrhea virus through regulating glycolysis/gluconeogenesis

**DOI:** 10.1080/01652176.2025.2570471

**Published:** 2025-10-13

**Authors:** Huihui Li, Xiaoyu Huang, Na Yuan, Lixian Wang, Lijun Shi

**Affiliations:** a State Key Laboratory of Animal Biotech Breeding, Institute of Animal Science, Chinese Academy of Agricultural Sciences, Beijing, China; b Institute of Industrial Technological Innovation, Beijing Vica Biotechnology Co., LTD, Beijing, China

**Keywords:** Chinese Min piglets, Yorkshire piglets, PEDV, transcriptome, IPEC-J2, *G6PC3* gene

## Abstract

Porcine epidemic diarrhea virus (PEDV) causes a highly contagious disease in pigs, and the intricacies of its host–pathogen interactions require further elucidation. Chinese Min piglets, known for their superior resistance to stress and disease, were compared with Yorkshire piglets to investigate breed-specific resistance mechanisms. We established PEDV infection models in both breeds and analyzed differences by assessing cytokine levels, viral loads, and histological changes in jejunal tissues. Transcriptomic analysis of jejunal tissues identified 5422 differentially expressed (DE) protein-coding genes (PCGs) and 1999 DE long non-coding RNAs (lncRNAs) between the two pig breeds. Functional annotation revealed that Yorkshire piglets exhibited upregulation of inflammatory and apoptotic pathways, whereas Chinese Min piglets displayed strong inflammatory responses and enhanced mucosal immunity. Notably, glucose-6-phosphatase catalytic subunit 3 (*G6PC3*) expression was significantly higher in Chinese Min piglets than in Yorkshire piglets. Knockdown of *G6PC3* in the intestinal porcine epithelial cell line J2 (IPEC-J2) resulted in increased PEDV replication and decreased expression of immune-related genes involved in the glycolysis/gluconeogenesis metabolism pathway. These findings highlight the distinct immune responses of Chinese Min and Yorkshire piglets to PEDV infection, and identify key PCGs and lncRNAs associated with PEDV immunity.

## Introduction

Porcine epidemic diarrhea virus (PEDV), measuring from 95 to 190 nm, belongs to the Alphacoronavirus genus (Duarte et al. 1993; Lin et al. 2016), and poses a serious global threat to the swine industry (Stevenson et al. 2013). PEDV-infected pigs typically exhibit symptoms such as atrophy, blunting, fusion, and loss of intestinal villi, which severely impair digestion and nutrient absorption (Sueyoshi et al. 1995; Jung et al. 2006; Jung and Saif 2015). The primary lesions in PEDV-infected pigs are localized in the jejunum and ileum, with the jejunum being the most severely affected (Li et al. 2012; Song and Park 2012; Zhang et al. 2020). Mutations in the PEDV genome, along with the challenges of inducing effective mucosal immunity, complicate the development of vaccines against emerging mutant strains (Lin et al. 2016). Therefore, identifying functional molecular markers to enhance resistance to PEDV is a promising alternative strategy.

Several genes, such as *IL-8*, *Bcl-2,* and *EGFR*, have been identified as playing important roles in PEDV infection (Xu et al. 2013; Yang et al. 2018). In addition, signaling pathways such as PI3K/AKT and mTOR have been implicated in resistance to PEDV (Shen et al. 2020). Proteomic analysis of 7-day-old piglets infected with PEDV strains of different virulence has revealed differential expression of antiviral molecules, such as Mx1, OAS1, and HSP family proteins (Li et al. 2016). Although some progress has been made in understanding the basic pathogenesis and immune response to PEDV infection, further investigation is needed to explore the differences among various breeds.

Chinese Min pigs are known for their superior resistance to stress and disease, exhibiting significantly higher proportions of CD4+ and CD8+ T lymphocytes than Landrace pigs (Guan et al. 2015). Additionally, their serum IgA and IgG concentrations are greater than those of Yorkshire pigs (Zhang et al. 2018). Resistance to PEDV infection among different pig breeds involves both shared and unique mechanisms, suggesting that studying diverse breeds could provide a broader spectrum of genetic markers associated with PEDV resistance.

In our previous studies, PEDV-infected pig models were established in both Yorkshire (Shi et al. 2024) and Chinese Min piglets (Li et al. 2023). We identified significant proteins and metabolites associated with PEDV infection in Yorkshire piglets, as well as key candidate protein-coding genes (PCGs), long non-coding RNAs (lncRNAs), and microRNAs (miRNAs) linked to PEDV infection in Chinese Min piglets (Li et al. 2023; Shi et al. 2024). In the present study, transcriptomic analysis of jejunal tissue from Yorkshire piglets infected with PEDV was performed, identifying candidate RNAs associated with PEDV infection in this breed. Furthermore, we conducted a comprehensive analysis comparing cytokine levels, relative viral load, and jejunal paraffin sections between Yorkshire and Chinese Min piglets. By comparing the expression profiles of key PCGs and lncRNAs, we explored the breed-specific differences in their immune responses and pathogenesis. Finally, to investigate the regulatory role of key gene glucose-6-phosphatase catalytic subunit 3 (*G6PC3*), we knocked down *G6PC3* in the porcine small intestinal epithelial cell line J2 (IPEC-J2) and examined its function in PEDV resistance.

## Materials and methods

### Animals

The construction method of PEDV-infected pig models was described in our previous studies (Li et al. 2023; Shi et al. 2024). Briefly, 12 Yorkshire piglets and 12 Chinese Min piglets, all deprived of colostrum, were fed BaoBaoLe milk (Centre Bio-tech (Wuhan) Co., Ltd, Wuhan, China). At three days of age, eight Yorkshire piglets and eight Chinese Min piglets were orally inoculated with 1 mL of PEDV (G2a mutant strain, 10 TCID_50_/mL). The remaining piglets, serving as controls, were given 1 mL of 0.9% saline (Beijing Epsilon Biotechnology Co., Ltd, Beijing, China). Based on survival status after infection, the piglets were divided into six groups: Yorkshire control group (YC), Chinese Min control group (MC), Yorkshire death group (YD), Chinese Min death group (MD), Yorkshire resistance group (YR), and Chinese Min resistance group (MR). In this study, 12 Yorkshire piglets were used for intra-breed comparisons, and their data were further compared with those of three groups of Chinese Min piglets reported previously (Li et al. 2023).

### ELISA for cytokine detection

Blood samples were collected from 12 Yorkshire piglets and 12 Chinese Min piglets into EDTA-K2 anticoagulant tubes and centrifuged at 1500 × g for 10 min to obtain serum. The serum samples were analyzed for Interleukin-8 (IL-8), Interleukin-17 (IL-17), Tumor Necrosis Factor-α (TNF-α), Interferon-γ (IFN-γ), Immunoglobulin A (IgA), Immunoglobulin G (IgG), and Diamine Oxidase (DAO) levels using enzyme-linked immunosorbent assay (ELISA) kits (Shanghai Preferred Bioscience and Technology Co. Ltd., Shanghai, China).

For the jejunum tissue analysis, one gram (g) of tissue was placed in a tube with 500 μL of physiological saline and two small steel beads. The mixture was homogenized with an ultrasonic oscillator, and then centrifuged at 1500 × g for 10 min. A 500 μL aliquot of the supernatant was collected and analyzed for secretory immunoglobulin A (sIgA) and secretory immunoglobulin G (sIgG) levels using ELISA kits (Shanghai Preferred Bioscience and Technology Co. Ltd., Shanghai, China).

### Evaluation of intestinal injury

In our previous research (Li et al. 2023; Shi et al. 2024), viral nucleic acids were extracted from the intestinal tissues (duodenum, jejunum, ileum, colon, cecum, and rectum) of PEDV-infected piglets, and PEDV copy numbers were quantified. Additionally, H&E sections of the intestines were prepared. In this study, we assessed intestinal injury between the two breeds by analyzing relative viral load, measuring villus length in intestinal H&E sections, and evaluating serum DAO levels.

### RNA extraction, library construction, and sequencing

Jejunal tissues of 12 Yorkshire piglets in three groups were ground by a freeze grinder (Beijing Held Technology Co., Ltd, Beijing) and subjected to RNA extraction using the TRlzol reagent (Invitrogen, Carlsbad, CA, USA). RNA libraries for mRNAs and lncRNAs were constructed and sequenced at Beijing Novogene Technology Co., Ltd. (Beijing, China) (Li et al. 2023).

### Quality control and identification of mRNAs and lncRNAs

After removal of low-quality reads, adapters, and poly-N sequences, genome assembly, mapping, and quantification were performed as described in a previous study (Li et al. 2023). Known mRNAs and lncRNAs were annotated based on the reference genome Sscrofa11.1. Novel lncRNAs were identified using the following criteria: exon count ≥ 2, transcript length > 200 bp, open reading frame (ORF) ≤ 300 bp, expression in at least two samples, and no overlap with annotated exons in the reference genome. Non-coding transcripts were confirmed by CPC2 (Kang et al. 2017), PLEK (Li et al. 2014), and CNCI (Sun et al. 2013).

For differential expression analysis, PCGs and lncRNAs were considered if their Fragments Per Kilobase of transcript per Million mapped reads (FPKM) were ≥ 0.01 in at least four samples. In addition, we conducted principal component analysis (PCA) using the ggplot2 package in R to assess sample reproducibility and clustering.

### Identification and functional annotation of differentially expressed (DE) molecules

DE protein-coding genes (PCGs) and lncRNAs were identified using the DESeq2 package in R, with thresholds of |log2FC| > 1 and adjusted *P*-value (adjusted) < 0.05. Heatmaps were generated to visualize expression patterns of DE molecules across groups.

Cis-target genes were defined as PCGs located within 100 kb upstream or downstream of DE lncRNAs, with predictions performed by BEDTools (Quinlan and Hall 2010). Trans-target PCGs were identified by Pearson correlation analysis (|correlation| > 0.95 and *P*-value < 0.05) using the Hmisc package in R.

Functional annotation for DE molecules was performed using the DAVID online platform, with the significant enrichments determined for Gene Ontology (GO) terms and Kyoto Encyclopedia of Genes and Genomes (KEGG) pathways (FDR-adjusted *P* < 0.05).

### Transfection of siRNAs targeting *G6PC3* and lncRNA *ENSSSCG00000055033*

IPEC-J2 cells were obtained from Huazhong Agricultural University (Wuhan, China). IPEC-J2 cells were cultured in Dulbecco’s Modified Eagle Medium (DMEM) supplemented with 10% fetal bovine serum (FBS) and 1% penicillin-streptomycin (Gibco, Grand Island, NY, USA) at 37 °C in a 5% CO_2_ incubator.

Two siRNAs targeting *G6PC3* (*G6PC3-504* and *G6PC3-761*) and two siRNAs targeting lncRNA *ENSSSCG00000055033* (*lncRNA-504* and *lncRNA-761*), along with a negative control (NC) siRNA, were synthesized by GenePharma (Shanghai, China). The sequences of the siRNAs are listed in Table S1. Approximately 1 × 10^6^ cells were seeded into six-well plates prior to transfection. When the cells reached approximately 80% confluence, the siRNAs were transfected into the cells using Lipofectamine^TM^ 3000 (Invitrogen, Carlsbad, CA, USA) according to the manufacturer’s instructions. Transfection efficiency was evaluated by real-time quantitative PCR (RT-qPCR) 48 h post-transfection. IPEC-J2 cells exhibiting high transfection efficiency were used for subsequent PEDV infection experiments.

### Effects of *G6PC3* on PEDV replication

IPEC-J2 cells with effective *G6PC3* knockdown and control cells were infected with PEDV G2a at a multiplicity of infection (MOI) of 0.1. After 2 h of incubation at 37 °C, the infection medium was replaced with 2 mL of standard culture medium. The PEDV N gene and five PCGs involved in the glutamate metabolic pathway (*ACSS1*, *ENO1*, *HK3*, *PKM*, and *PFKL*) were quantified by qRT-PCR at 24 h post-infection (hpi). The expression of five cytokine genes *(IL1B, IL6, IL18, IFN-α*, and *IFN-β*) was also analyzed in the *G6PC3* knockdown and control groups following PEDV infection.

### Real-time quantitative PCR (RT-qPCR)

Total RNA was extracted from cells using an RNA extraction kit (Nanjing Novezan Biotechnology Co., Ltd., Nanjing, China), and reverse-transcribed into cDNA with the PrimeScript^™^ RT reagent Kit (TaKaRa Bio, Kusatsu, Japan). qRT-PCR was performed using TB Green^™^ Premix Ex Taq^™^ (Tli RNase H Plus) on a QuantStudio^™^ 7 Flex Real-Time PCR System (Applied Biosystems, Carlsbad, CA, USA). The cycling protocol included an initial denaturation at 95 °C for 30 s; followed by 40 cycles of 95 °C for 5 s, 60 °C for 34 s; and a final melt curve stage consisting of 95 °C for 15 s, 60 °C for 1 min, and 95 °C for 15 s. Gene expression levels were normalized to *GAPDH*. Primer sequences (Table S1) were designed using Primer3 and synthesized by BGI Genomics Co., Ltd. (Shenzhen, China).

### Cell viability assay

Cell viability after PEDV infection and *G6PC3* knockdown in IPEC-J2 cells was assessed using the Cell Counting Kit-8 (CCK-8, Bioman Technology Co., Ltd., Beijing, China). Briefly, 5000 cells per well from the PEDV-free control group and cells infected with PEDV for 24 h were seeded into 96-well plates. After 12 h of culture, 10 μL of CCK-8 reagent was added to each well, followed by incubation for 2 h. Absorbance at 450 nm was measured using a microplate reader to compare viability across treatment groups. Following transfection with *G6PC3* siRNA for 24 h, the same procedure was applied to evaluate cell viability in NC and siRNA groups.

### 5-ethynyl-2′-deoxyuridine (EdU) assay

Cell proliferation was assessed using the BeyoClick EdU Cell Proliferation Kit (Beyotime, Shanghai, China). Comparisons were made between PEDV-infected cells (24 hpi) and uninfected controls, as well as between NC-transfected cells and *G6PC3* siRNA-transfected cells (24 h post-transfection). Cells were incubated with 10 μM EdU for 2 h, stained with Azide 555 (red) and Hoechst 33342 (blue), and visualized under a fluorescence microscope (Leica Microsystems, Wetzlar, Germany) at 200× magnification. EdU-positive cells were quantified using ImageJ software.

### Immunofluorescence detection of PEDV

The culture medium was removed, and the cells were rinsed three times with pre-warmed 1× PBS. The cells were then fixed with 4% paraformaldehyde for 10 min and permeabilized with 0.25% Triton X-100 (Beyotime, Shanghai, China) for 10 min. Following permeabilization, the samples were blocked with 5% bovine serum albumin (BSA) for 2 h. Primary antibodies (1:100 dilution) were applied and incubated at 4 °C for 12 h. After washing, secondary antibodies (1:200 dilution) were added and incubated at room temperature (RT) in the dark for 2 h. Nuclei were counterstained with DAPI (Thermo Fisher Scientific, Waltham, MA, USA) for 10 min at RT. Finally, immunofluorescence images were acquired using a Leica DM300 fluorescence microscope (Leica Microsystems, Wetzlar, Germany) at 200× magnification. ImageJ software (version 1.46r) was used to statistically analyze the proportion of PEDV fluorescence area relative to the total cell area.

### Western blot analysis of PEDV N protein

IPEC-J2 cells were lysed in RIPA buffer (Beyotime, Shanghai, China) supplemented with PMSF protease inhibitor (Sigma, St. Louis, MO, USA) at a ratio of 1:100. Protein concentration was determined using the BCA assay kit (Beyotime, Shanghai, China). Subsequently, 5× SDS-PAGE Protein Sample Buffer (Beyotime, Shanghai, China) was added to the protein samples at a ratio of 1:4, and the mixture was denatured by heating at 95 °C for 5 min. The denatured protein samples were separated on a 10% SDS-PAGE gel and transferred onto a polyvinylidene difluoride (PVDF) membrane (Beyotime, Shanghai, China). Membranes were blocked with 5% skim milk containing 0.05% Tween 20 for 1 h at RT. The PVDF membranes were incubated with the primary antibody against PEDV N (Shanghai Youlong Biotechnology Co., Ltd., Shanghai, China, Catalog Number: DAO111, 1:1000 dilution) for 12 h at 4 °C. A β-actin antibody (Beyotime, Shanghai, China, Catalog Number: AF5003, 1:10,000 dilution) was used under the same conditions as the loading control.

After primary antibody incubation, HRP-labeled Goat Anti-Mouse IgG (H + L) secondary antibody (Beyotime, Shanghai, China) was applied at a 1:5000 dilution and incubated for 1 h at RT. Protein bands were visualized using BeyoECL Moon chemiluminescence reagent (Beyotime, Shanghai, China) according to the manufacturer’s instructions. Band intensities were quantified using ImageJ software (version 1.46r), and protein expression levels were normalized to β-actin.

### Statistical analysis

All experiments were performed in triplicate. Statistical significance for cytokine levels, DAO levels, PEDV expression, villus length, and quantitative data was evaluated using independent-sample *t*-tests (*P* < 0.05). Graphs were generated using GraphPad Prism 8 software, and error bars represent standard deviations (SDs).

## Results

### Cytokine differences between Yorkshire and Chinese Min piglets infected with PEDV

**Figure 1.**
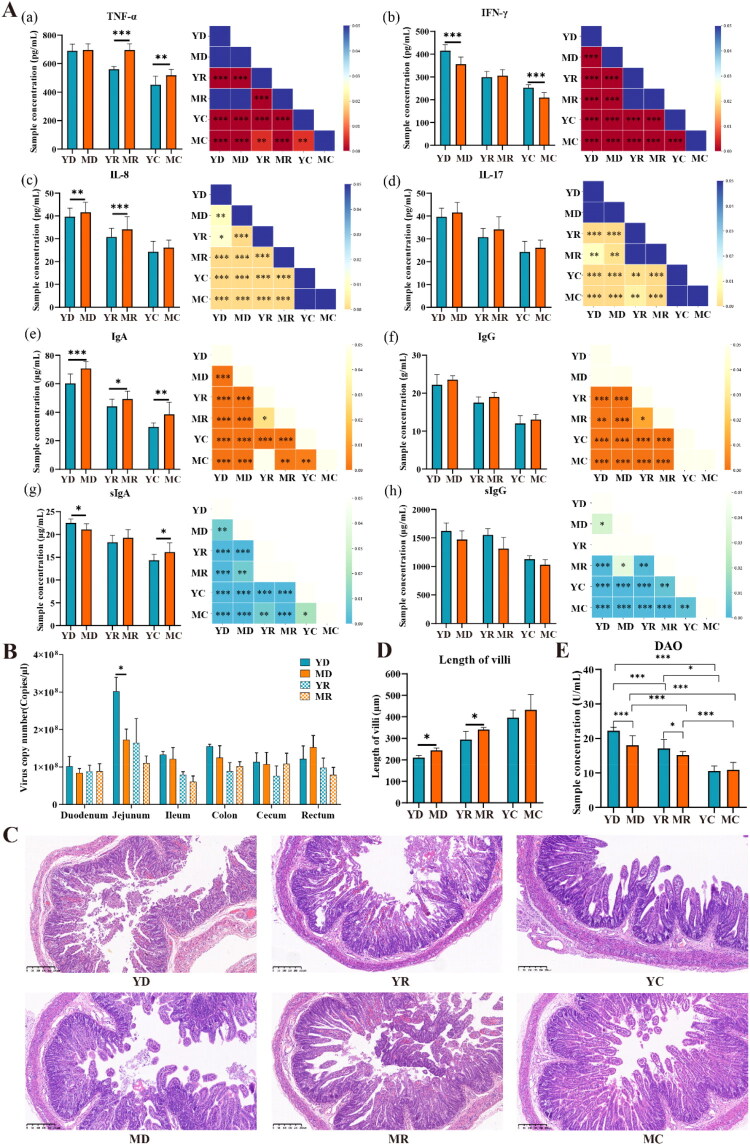
Phenotypic measurements of Yorkshire and Chinese Min piglets, including Yorkshire death group (YD), Yorkshire resistance group (YR), Yorkshire control group (YC), Chinese Min death group (MD), Chinese Min resistance group (MR), and Chinese Min control group (MC). (A) ELISA analysis showed significant differences in serum and intestinal cytokine levels between Yorkshire and Chinese Min groups. Bar charts depict cytokine levels in different groups and the lower triangular plot shows the *P-value*s from t-tests among these groups. (**B)** PEDV copy numbers in the small intestine of Yorkshire and Chinese Min piglets infected with PEDV. (**C)** Representative jejunal sections from Yorkshire and Chinese Min piglets, showing severe congestion and stasis in interstitial vessels (magnification, 100×). (**D)** Villus length in Yorkshire and Chinese Min piglets. **(E)** Serum DAO levels in Yorkshire and Chinese Min piglets. * indicates *P* < 0.05, ** indicates *P* < 0.01, and *** indicates *P* < 0.001.

In both Yorkshire and Chinese Min piglets, PEDV infection resulted in higher levels of IFN-γ, IL-8, IL-17, IgA, IgG, and sIgA in the death group than in the resistance group, and in the resistance group than in the control group (Figure 1A, P < 0.05). Within the Yorkshire piglet group, no significant difference in sIgG levels was observed between the death and resistance groups; however, both groups exhibited higher sIgG levels than the control group (Figure 1A, P < 0.05). In Chinese Min piglets, TNF-α levels were significantly higher in the death and resistance groups than in the control group (Figure 1A, P < 0.05), but did not differ between the death and resistance groups. Furthermore, Yorkshire piglets exhibited a stronger T cell-mediated response, characterized by higher IFN-γ and sIgG levels than Chinese Min piglets (Figure 1A, P < 0.05). In contrast, Chinese Min piglets demonstrated higher levels of TNF-α, IL-8, IgA, and sIgA compared to Yorkshire piglets, suggesting a greater reliance on early inflammatory and mucosal defenses (*P* < 0.05). There were no significant differences in IL-17 and IgG levels between the two breeds, indicating comparable roles for these factors in the PEDV immune response.

### Severe jejunal tissue damage caused by PEDV

The relative expression of PEDV in the duodenum, jejunum, ileum, colon, cecum, and rectum of Yorkshire and Chinese Min piglets in the death group (YD and MD) was significantly higher than that in the resistance group (YR and MR), with the jejunum showing the highest levels (Figure 1B) (Li et al. 2023; Shi et al. 2024). Notably, PEDV load in jejunal tissues differed significantly between the YD and MD groups (Figure 1B, P < 0.05). Intestinal villus length was significantly reduced in the YD and the YR groups compared with the corresponding Chinese Min pig groups (Figure 1C,D *P* < 0.05). Additionally, DAO levels were markedly higher in the YD and YR groups compared with the MD and MR groups (Figure 1E, *P* < 0.05). No significant differences in villus length or DAO levels were observed between YC and MC groups (*P* > 0.05).

### Jejunum transcriptomic profiles and lncRNA target gene analysis in Yorkshire piglets

**Figure 2.**
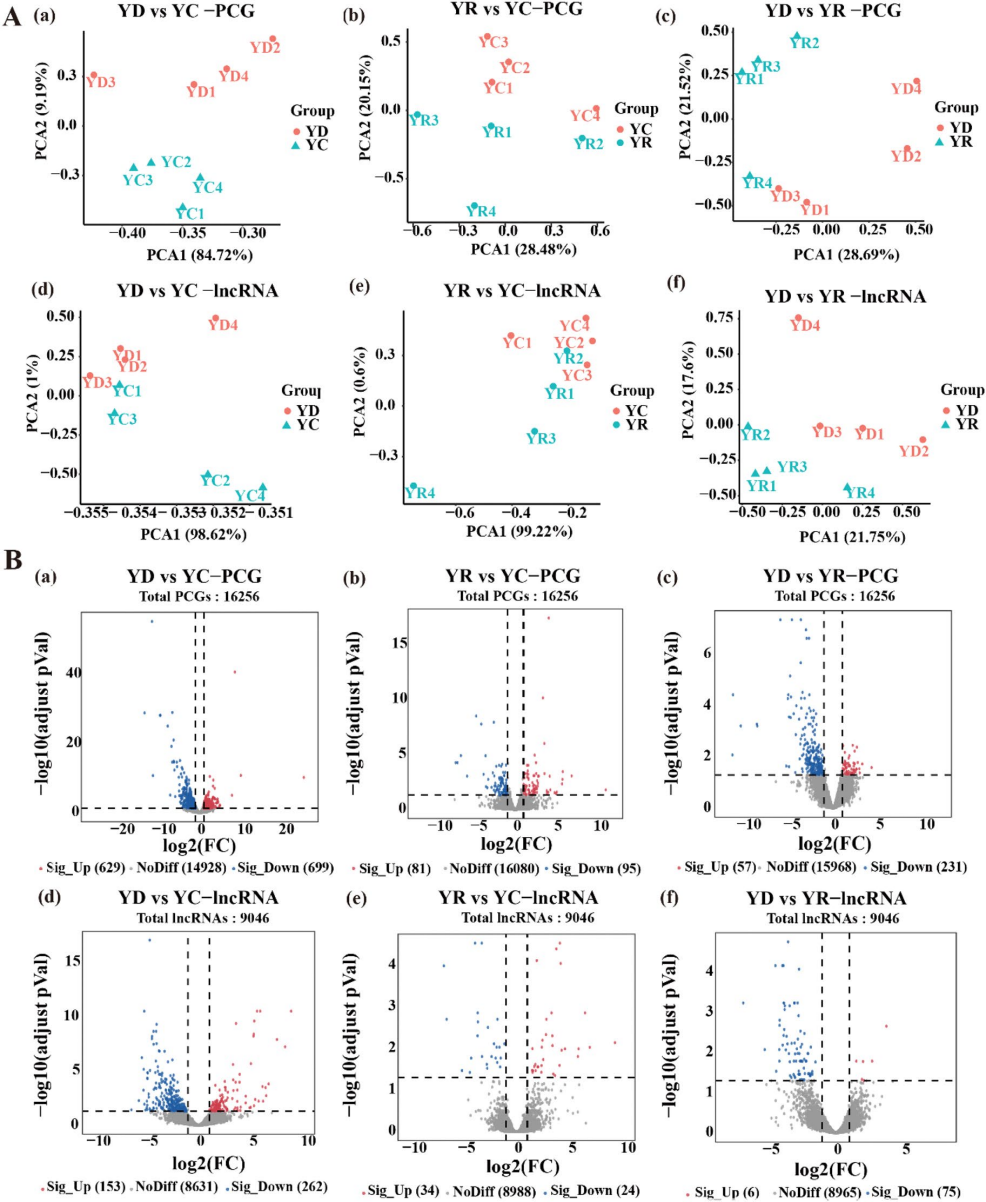
Transcriptome analysis of jejunal tissue in Yorkshire piglets infected with PEDV. PCG: Protein-coding gene. lncRNA: Long non-coding RNA. YD: Yorkshire death group. YR: Yorkshire resistance group. YC: Yorkshire control group. (A) PCA plots showing clear separation of the PCG and lncRNA data for the YD, YR, and YC groups. (B) Volcano plots showing differentially expressed (DE) analysis. Log_2_(FC): the logarithm to the base 2 of the fold change in gene expression. -log10(adjust pVal): Negative log base 10 of the Bonferroni-adjusted *P*-value. Sig_up: upregulated DE molecules. NoDiff: No DE molecules. Sig_down: downregulated DE molecules. Threshold lines indicate Bonferroni-adjusted *P* < 0.05 and |log_2_(FC)| > 1.

In this study, a total of 16,256 PCGs and 9046 lncRNAs (3398 known and 5648 novel) were identified in Yorkshire piglets. PCA demonstrated clear distinctions between the three groups (YC, YD, and YR) of Yorkshire piglets, with strong sample reproducibility (Figure 2A). DE analysis revealed 8175 DE PCGs and 3627 DE lncRNAs (Figure 2B). For PCGs, 1328 DE PCGs were identified in the YD versus YC (629 upregulated and 699 downregulated) (Figure 2B(a)); 176 DE PCGs in YR versus YC (81 upregulated and 95 downregulated) (Figure 2B(b)); and 288 DE PCGs in YD versus YR (57 upregulated and 231 downregulated) (Figure 2B(c)). For lncRNAs, 415 DE lncRNAs were identified in YD vs. YC (153 upregulated and 262 downregulated) (Figure 2B(d)); 58 DE lncRNAs in YR versus YC (34 upregulated and 24 downregulated) (Figure 2B(e)); and 81 DE lncRNAs in YD versus YR (6 upregulated and 75 downregulated) (Figure 2B(f)). These findings revealed significant transcriptional changes in the YD group, particularly marked down-regulation of PCGs and lncRNAs compared with the YC group. In contrast, the relatively balanced expression in the YR group suggested distinct immune mechanisms underlying PEDV resistance.

In the YD versus YC comparison, upregulated lncRNAs were predicted to target 2628 genes, while downregulated lncRNAs were associated with 977 PCGs. In YR versus YC, 876 and 757 genes were linked to upregulated and downregulated lncRNAs. In YD versus YR, upregulated lncRNAs targeted 1345 genes, whereas downregulated lncRNAs targeted 434 genes.

### Immune pathway mobilization following PEDV infection in Yorkshire piglets

Functional enrichment analysis of DE PCGs and lncRNAs across YD, YR, and YC groups revealed distinct pathway activations. In the comparison between YD and YC, the upregulated DE PCGs in YD were mainly enriched in human diseases, including prion disease, Alzheimer’s disease, and diabetic cardiomyopathy. In YR versus YC, upregulated DE PCGs were associated with inflammatory immune pathways, including the IL-17, NF-κB , and TNF signaling pathways. In contrast, DE PCGs highly expressed in YC compared with both YD and YR were mainly enriched in metabolic and synaptic processes, including protein digestion and absorption, glycosphingolipid biosynthesis (globo and isoglobo series), and amino acid metabolism. In YD versus YR, upregulated DE PCGs were linked to cardiovascular system-related functions, including the positive regulation of vascular smooth muscle cell migration, regulation of blood vessel diameter, and positive regulation of vascular smooth muscle cell proliferation. Conversely, downregulated DE PCGs were enriched in metabolic mechanisms and immune processes, including PPAR signaling pathway, fatty acid metabolism, glycolysis/gluconeogenesis, antigen processing and presentation, natural killer cell-mediated cytotoxicity, and Th17 cell differentiation. Enrichment analysis of lncRNA targets reflected similar trends.

### Jejunum transcriptomic differences between two breeds

**Figure 3.**
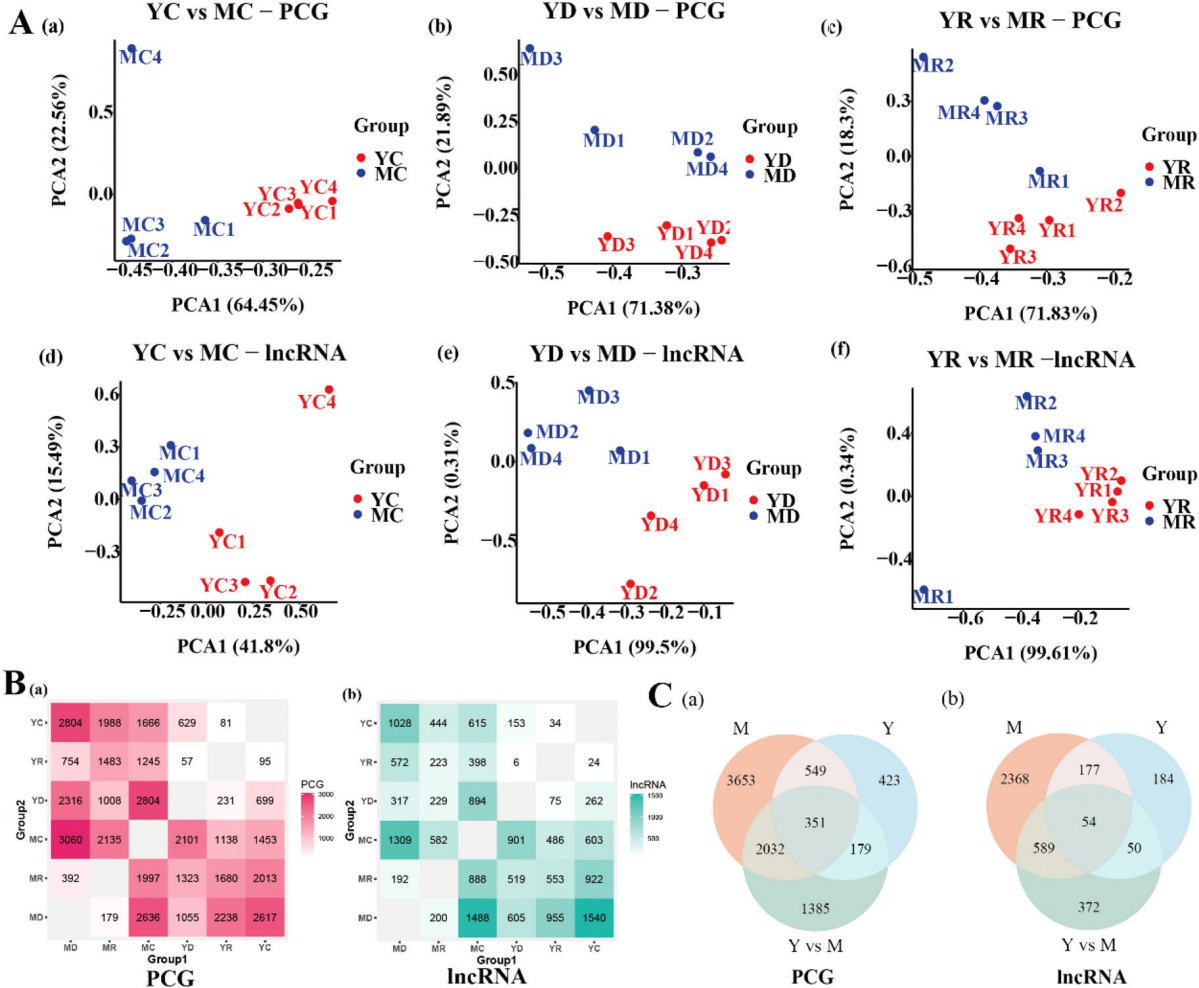
Comparative transcriptomic analysis of jejunal tissues in Yorkshire and Chinese Min piglets. PCG: Protein-coding gene. lncRNA: Long non-coding RNA. YD: Yorkshire death group; YR: Yorkshire resistance group; YC: Yorkshire control group; MD: Chinese Min death group; MR: Chinese Min resistance group; MC: Chinese Min control group. (A) PCA plots showing clear separation of the PCG and lncRNA data across all the groups. (B) Counts of differentially expressed (DE) PCGs and lncRNAs between different groups. The top left of each box indicates the number of upregulated DE molecules, and the bottom right indicates the number of downregulated DE molecules. (C) Venn diagram showing the intersection of DE molecules between and within breeds. Y: DE molecules from comparisons within Yorkshire piglets (YD vs. YC and YR vs. YC); M: DE molecules from comparisons within Chinese Min piglets (MD vs. MC and MR vs. MC); Y versus M: DE molecules from inter-breed comparisons (YD vs. MD and YR vs. MR).

We performed three comparisons (YC vs. MC, YD vs. MD, and YR vs. MR) across PCGs and lncRNAs, and PCA revealed clear separation among groups (Figure 3A). In YC vs. MC, 3119 DE PCGs were identified, including 1453 upregulated and 1666 downregulated (Figure 3B(a)). In YD versus MD, 3371 DE PCGs were identified, of which 1055 were upregulated and 2316 were downregulated (Figure 3B(a)). In YR versus MR, 3163 DE PCGs were identified, with 1680 upregulated and 1483 downregulated (Figure 3B(a)). For lncRNAs, 2568 DE transcripts were detected in the YC versus MC, including 1540 upregulated and 1028 downregulated (Figure 3B(b)). In YD versus MD, 922 DE lncRNAs were identified, of which 605 were upregulated and 317 were downregulated, respectively (Figure 3B(b)). In YR versus MR, we detected 1527 DE lncRNAs, with 955 upregulated and 572 downregulated (Figure 3B(b)).

In YD versus MD, 1050 target genes were linked to upregulated lncRNAs, and 600 to downregulated lncRNAs. In YR versus MR, upregulated lncRNAs were associated with 1448 target genes, and downregulated lncRNAs were associated with 860 target genes. In YC versus MC, upregulated lncRNAs were associated with 1506 target genes, whereas downregulated lncRNAs were linked to 2074 target genes.

### Metabolic and disease resistance differences between Yorkshire and Chinese Min piglets

Numerous DE molecules in the YC versus MC highlighted differences in immunity, metabolism, and disease susceptibility between Yorkshire and Chinese Min piglets. In the YC group, upregulated molecules were primarily associated with immune responses and viral interactions, such as cytokine-cytokine receptor interaction and viral protein interaction with cytokine and cytokine receptor. In contrast, upregulated molecules in the MC group were involved in metabolic pathways and epithelial tight junction-related pathways, such as fructose and mannose metabolism, PPAR signaling pathway, and focal adhesion. DE PCGs and lncRNAs hinted at the genetic underpinnings of disease predisposition and resilience for the two pig breeds.

In YD versus MD, the enriched GO terms and KEGG pathways highlighted breed-specific responses. The pathways enriched with upregulated DE PCGs in YD were related to cellular processes and disease-related pathways, such as spliceosome, oxidative phosphorylation, herpes simplex virus 1 infection, and diabetic cardiomyopathy. Conversely, the pathways enriched with upregulated DE PCGs in MD were primarily focused on signal transduction and metabolism-related pathways, such as ECM-receptor interaction, focal adhesion, PI3K-Akt signaling pathway, JAK-STAT signaling pathway, pentose phosphate pathway, and biosynthesis of amino acids. In YR versus MR, enrichment analysis indicated distinct immune and metabolic responses. The pathways enriched by upregulated DE PCGs in YR were highly correlated with disease and oxidative stress, including COVID-19, Parkinson’s disease, oxidative phosphorylation and chemical carcinogenesis - reactive oxygen species. In MR, upregulated DE PCGs were mainly enriched in energy metabolism processes and immune and inflammatory responses, such as glycerophospholipid metabolism, glutathione metabolism, pentose phosphate pathway, glycolysis/gluconeogenesis, ECM-receptor interaction and focal adhesion.

### Insights into breed-specific mechanisms of PEDV resistance

The DE molecules identified between Yorkshire and Chinese Min piglets may only partially reflect PEDV resistance, as many differences are likely attributable to inherent breed-specific characteristics. To better delineate PEDV resistance-associated DE molecules, we defined three sets of DE molecules from the specific comparisons: Group Y comprised DE molecules from intra-breed comparisons within Yorkshire piglets (death group vs. control group, and resistance group vs. control group). Group M comprised DE molecules from corresponding intra-breed comparisons within Chinese Min piglets, (death group vs. control group, and resistance group vs. control group); and Group Y versus M, comprised DE molecules from inter-breed comparisons (YD vs. MD, and YR vs. MR). The overlap among these three sets was examined (Figure 3C). The intersection of Groups Y and M contained 900 PCGs and 231 lncRNAs, suggesting a cross-breed, conserved PEDV response mechanism. For Chinese Min piglets, the overlap of Groups M and Y versus M contained 2383 PCGs and 643 lncRNAs, while the corresponding intersection for Yorkshire piglets (Groups Y and Y vs. M) contained 530 PCGs and 104 lncRNAs. These DE molecules may underlie the observed difference in resistance between breeds. Importantly, molecules that were significantly DE across all three sets likely represent the core PEDV response, highlighting their potential key roles in both Yorkshire and Chinese Min piglets. The intersection shared by all three groups yielded 351 PCGs and 54 lncRNAs.

Focusing on the 2032 PCGs and 589 lncRNAs uniquely associated with PEDV resistance in Chinese Min piglets, GO enrichment analysis highlighted processes such as the negative regulation of chronic inflammatory response to antigenic stimulus, negative regulation of MHC class II biosynthetic process, negative regulation of cytokine production involved in immune response, and immune response. KEGG pathway analysis revealed significant enrichments in the PI3K-Akt signaling pathway, pathways in cancer, carbon metabolism, and metabolic Pathways. Notably, *PIK3CD* and *G6PC3* were involved in both PI3K-Akt and metabolic pathways. Importantly, *G6PC3* expression was markedly higher in Chinese Min piglets (both death and resistance groups) than in Yorkshire piglets. Further, *G6PC3* expression was upregulated in the MR compared to the MC. These findings implicate that *G6PC3* might be a key candidate for PEDV resistance.

### Negative impact of PEDV on IPEC-J2 proliferation

**Figure 4.**
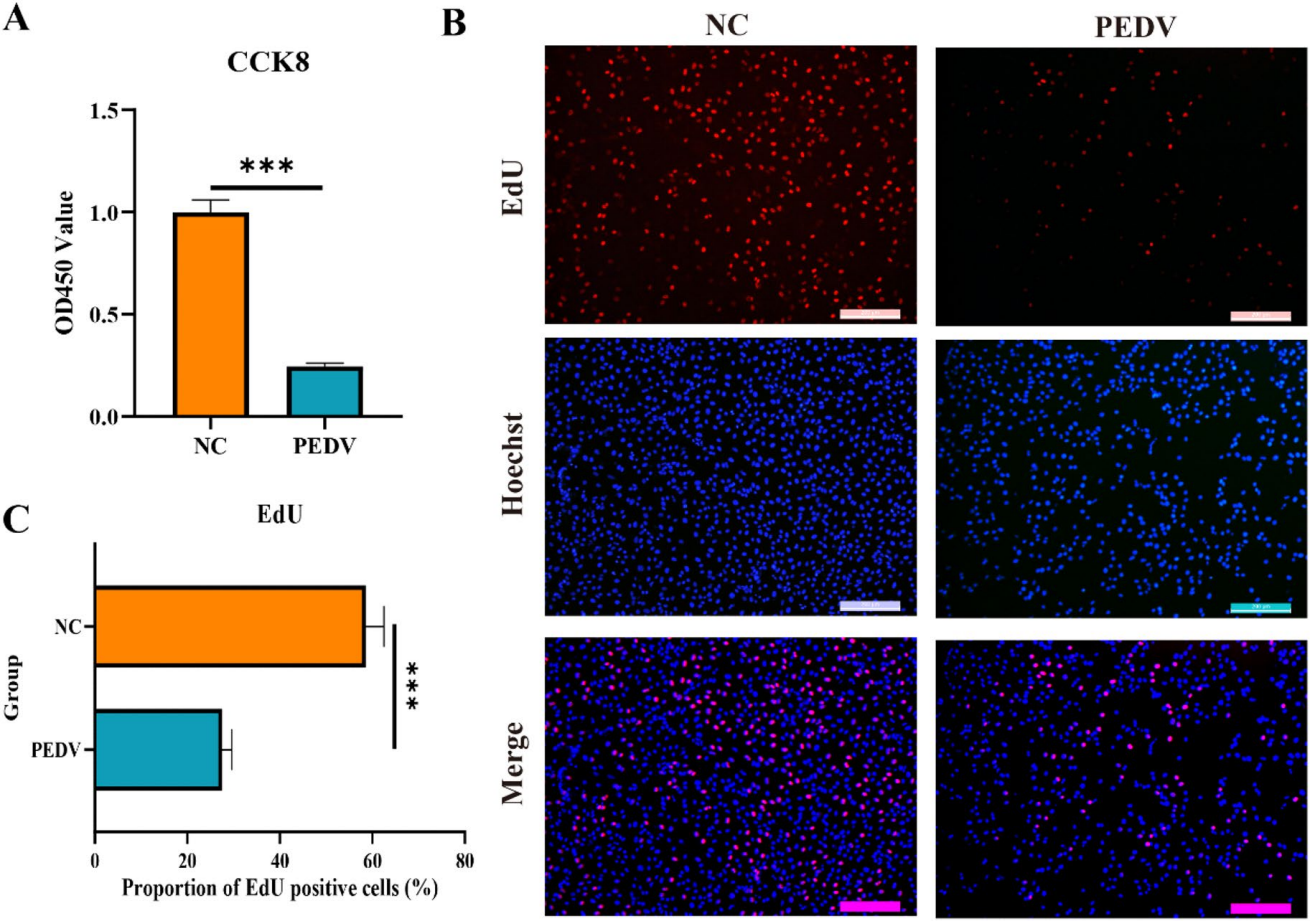
Effects of PEDV infection on the biological function of IPEC-J2 cells. (A) CCK-8 assay showing that PEDV infection significantly reduced cell proliferation of IPEC-J2 compared to the uninfected control group. (B, C) EdU assay showing a significant reduction in proliferation capacity of PEDV-infected IPEC-J2 cells compared with the uninfected control group.

In the *in vitro* model, cytopathic effects of PEDV were observed in IPEC-J2 cells. CCK-8 and EdU assays demonstrated that PEDV infection significantly inhibited IPEC-J2 proliferation (Figure 4). These findings are consistent with the intestinal tissue damage and increased mucosal transparency observed in infected pigs, providing further insight into the pathological mechanisms of PEDV.

### *G6PC3* contributes to host resistance against PEDV by modulating glycolysis/gluconeogenesis pathway

A deficiency in *G6PC3* leads to metabolic dysregulation and impaired bacterial clearance through mTOR signaling, potentially exacerbating inflammatory responses (Bolton et al. 2022). *G6PC3* participates in various signaling pathways, including metabolic pathways, PI3K-Akt signaling pathway, AMPK signaling pathway, glycolysis/gluconeogenesis, insulin signaling pathway, galactose metabolism, and FoxO signaling pathway. Enrichment analysis of DE PCGs in YD versus YR and YR versus MR indicated activation of glycolysis/gluconeogenesis pathways. Glycolytic products are critical for fulfilling the energy and biosynthesis demands of immune cells such as T cells, B cells, macrophages, and dendritic cells during immune responses (Wang and Green 2012; Buck et al. 2015; O’Neill et al. 2016; O’Neill and Pearce 2016; Muri and Kopf 2021). However, the precise mechanisms by which *G6PC3* contributes to PEDV resistance remain unclear.

**Figure 5.**
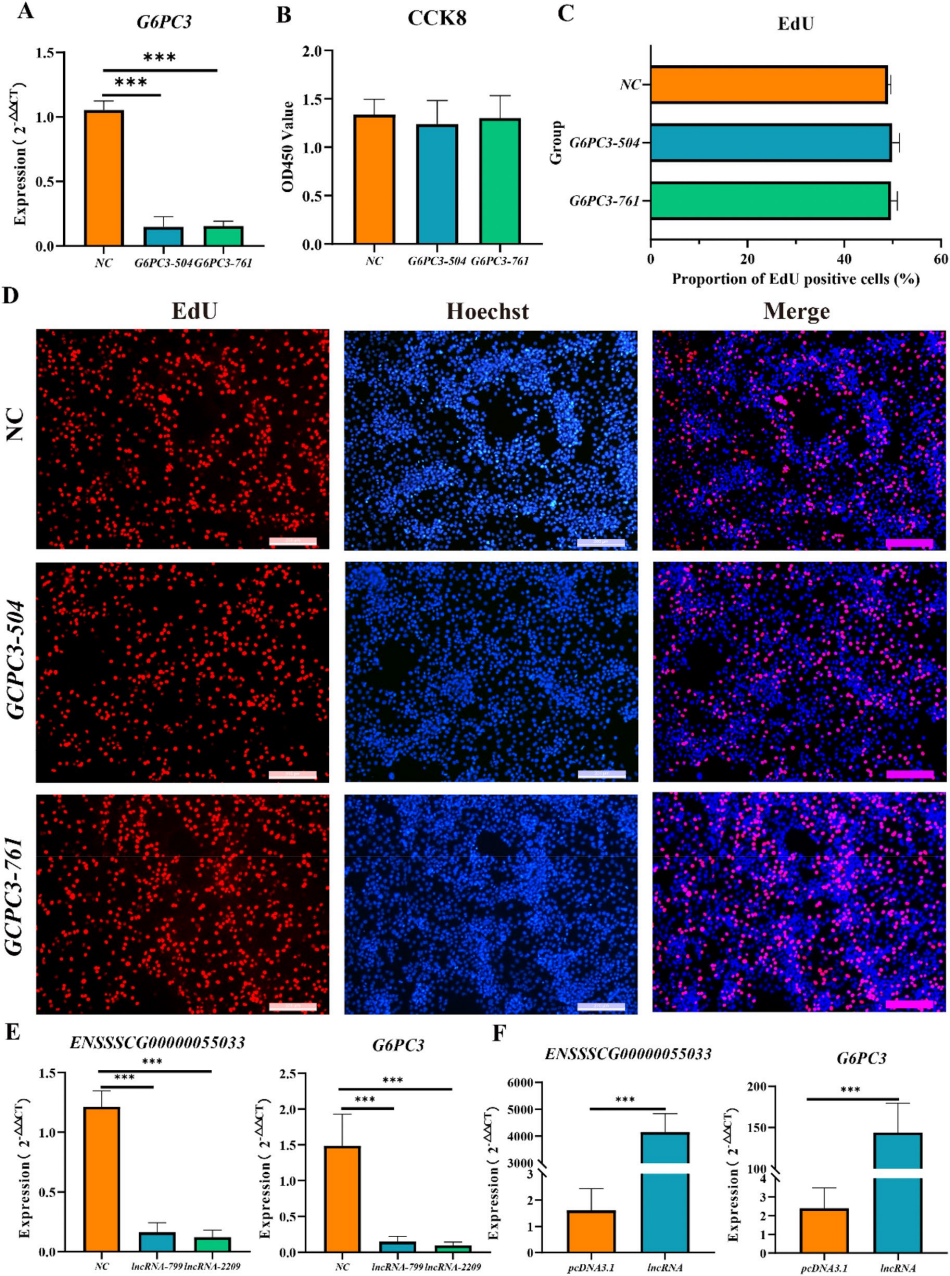
Effects of *G6PC3* knockdown on the biological function of IPEC-J2 cells. (A) Knockdown of *G6PC3* significantly reduced its expression in IPEC-J2 cells. (B) CCK-8 assay showing no significant difference in proliferation between *G6PC3* knockdown cells and controls. (C, D) EdU assay results similarly showing no change in the proliferation capacity of IPEC-J2 cells after *G6PC3* knockdown. (E) Knockdown of lncRNA *ENSSSCG00000055033* significantly reduced the expression of both *ENSSSCG00000055033* and *G6PC3*. (F) Over-expression of lncRNA *ENSSSCG00000055033* significantly increased the expression of both *ENSSSCG00000055033* and *G6PC3.*

To further clarify the function of *G6PC3* in PEDV replication, we performed *G6PC3* knockdown in IPEC-J2 cells using siRNAs (*G6PC3-504* and *G6PC3-761*). RT-qPCR analysis showed knockdown efficiency of 85.32% and 85.92%, respectively (Figure 5A, *P* < 0.001). Moreover, cell proliferation assays (CCK-8 and EdU) revealed no significant differences between *G6PC3* siRNA–treated cells and NC siRNA controls (Figure 5B–D), ensuring that *G6PC3* knockdown did not compromise cell viability.

**Figure 6.**
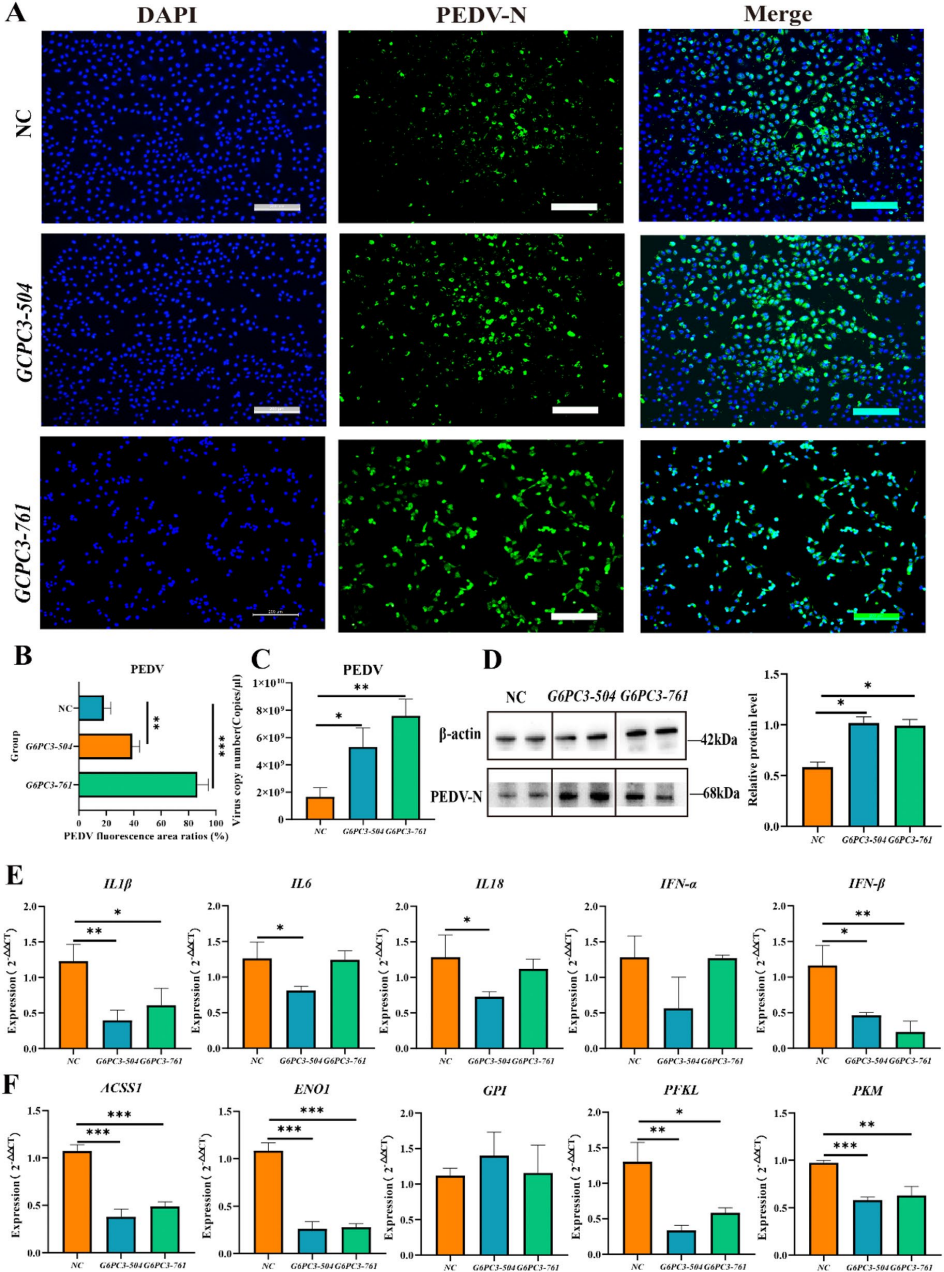
Effects of *G6PC3* knockdown on PEDV replication and related gene expression in IPEC-J2. * indicates *P* < 0.05, ** indicates *P* < 0.01, and *** indicates *P* < 0.001. (A) IFA showing stronger PEDV fluorescence signals in *G6PC3* knockdown groups compared to the control group, indicating elevated viral replication. (B) Semi-quantitative analysis of PEDV fluorescence area relative to total cell area, showing significantly higher ratios in *G6PC3-504* and *G6PC3-761* groups compared with the NC group. (**C)** RT-qPCR showing significantly increased expression of the PEDV N gene in *G6PC3* knockdown cells. (D) Western blot confirming increased protein levels of PEDV N in the *G6PC3* knockdown groups. (E, F) Knockdown of *G6PC3* significantly reduced expression of key cytokine genes (*IL-1β, IL-6, IL-18*, and *IFN-β*) and glycolysis/gluconeogenesis pathway related genes (*ACSS1*, *ENO1*, *PFKL*, and *PKM*).

PEDV replication was evaluated by IFA, qRT-PCR, and WB (Figure 6A–D). IFA results showed that PEDV fluorescence intensity in both the *G6PC3-504* and *G6PC3-761* groups was significantly higher than in the NC group (Figure 6A,B, *P* < 0.05). Knockdown of *G6PC3* led to significant increases in PEDV N gene expression at both the RNA and protein levels (Figure 6C, D, *P* < 0.05). Moreover, the expression of key immune response genes, including *IL1β*, *IL6*, *IL18*, and *IFN-β*, was significantly reduced in *G6PC3*-knockdown cells following infection (Figure 6E, *P* < 0.05).

Further investigation of the glycolysis/gluconeogenesis metabolism pathway revealed downregulated expression of key genes, including *ACSS1*, *ENO1*, *PKM*, and *PFKL*, in *G6PC3*-knockdown cells (Figure 6F, *P* < 0.05). These results suggested that *G6PC3* contributes to PEDV resistance by modulating glycolysis and immune responses. The elevated expression of *G6PC3* in Chinese Min piglets, which exhibited reduced phenotypic damage upon PEDV infection, further supports its potential role in enhancing resistance to PEDV.

LncRNA *ENSSSCG00000055033* was predicted to act as a trans-regulator of *G6PC3* based on transcriptome analysis. To validate this interaction, we performed interference and overexpression experiments to modulate lncRNA *ENSSSCG00000055033* expression and subsequently examined *G6PC3* levels. RT-qPCR confirmed the efficiency of both knockdown and overexpression, with significantly reduced and elevated lncRNA expression, respectively (Figure 5E and F, *P* < 0.001). In the interference group, *G6PC3* expression decreased by an average of 11-fold compared with the NC group (Figure 5E, *P* < 0.001). Conversely, in the overexpression group, *G6PC3* expression increased by approximately 59-fold relative to the NC group (Figure 5F, *P* < 0.001). These findings demonstrate that lncRNA *ENSSSCG00000055033* positively regulates *G6PC3* expression as a trans-acting factor.

## Discussion

In the present study, we successfully established PEDV-infected piglet models in Yorkshire and Chinese Min piglets, revealing distinct immune responses between them. Yorkshire piglets primarily relied on T cell-mediated immunity, whereas Chinese Min piglets exhibited stronger mucosal immune responses. Intestinal damage and viral load were significantly more severe in Yorkshire piglets compared to Chinese Min piglets. Transcriptomic analysis of jejunal tissues identified numerous DE molecules, indicating breed-specific expression patterns in response to PEDV infection. Using an *in vitro* interference model, we demonstrated that PEDV infection markedly inhibited IPEC-J2 proliferation and induced apoptosis, consistent with the pathological intestinal changes observed *in vivo*. Functional validation further revealed that knockdown of *G6PC3* significantly enhanced PEDV replication by modulating the glycolysis/gluconeogenesis metabolism pathway.

Significant increases in cytokine expression were observed in infected groups compared with control groups, indicating that both Yorkshire and Chinese Min piglets mounted robust immune responses to PEDV infection by mobilizing diverse cytokines. IFN-γ, which enhances the expression of immunosuppressive markers in tumor-associated lymphatic endothelial cells (LEC), plays a role in promoting T cell-mediated tumor destruction and inhibiting metastasis (Garnier et al. 2022). IgG and IgA serve as key effectors in humoral immunity (Schroeder and Cavacini 2010). sIgG and sIgA are tightly linked to mucosal epithelial cells, forming an immune barrier to prevent pathogen invasion (Chintalacharuvu and Morrison 1997). The elevated IFN-γ and sIgG levels observed in both the control and death groups of Yorkshire piglets vs. Chinese Min piglets suggested that these pigs relied more on cell-mediated immune responses, particularly T cell functionality, to combat PEDV infection. IL-8 promotes neutrophil and T cell chemotaxis (Chatzopoulou et al. 2019), and TNF-α enhances lymphocyte infiltration to the infection site, playing a crucial role in the early antiviral response (Calcinotto et al. 2012). Chinese Min piglets might more effectively utilize inflammatory responses and mucosal immunity, as evidenced by their higher levels of IL-8, TNF-α, IgA and sIgA. These elevated levels might contribute to better prevention of pathogen invasion and spread. IL-17 plays a pivotal role in immune defense and tissue repair processes (Zenobia and Hajishengallis 2015). Between the two breeds, no significant difference in IL-17 and IgG levels was shown, indicating a similar role in PEDV response for Yorkshire and Chinese Min piglets.

PEDV invades pig intestinal tissues, leading to decreased intestinal thickness, altered macromolecular permeability, and disrupted development of intestinal epithelial cells (Sun et al. 2012; Jung and Saif 2015; Schweer et al. 2016). Elevated serum DAO levels serve as an indicator of impaired intestinal mucosal barrier function, implying increased intestinal permeability and potential damage to the integrity and maturity of the mucosa. (Tan et al. 2022). The reduction in intestinal villus length observed upon PEDV infection is associated with damage to intestinal barrier function. Yorkshire piglets demonstrated more severe structural damage and functional impairment in their intestines compared to Chinese Min piglets, which were able to maintain better intestinal architecture and function under comparable infection conditions.

Transcriptomic profiling of Yorkshire piglets in this study provided insights into the molecular mechanisms underlying their response to PEDV infection. We identified 16,256 PCGs, 9046 lncRNAs, and 920 miRNAs, with DE analysis revealing distinct gene expression patterns and pathway activation among the YD, YR, and YC groups. In YD versus YC, upregulated DE PCGs were mainly enriched in inflammatory responses and cell apoptosis, suggesting that YD exhibited a heightened immune response to PEDV infection. In YR versus YC, upregulated DE PCGs were associated with inflammatory immune pathways, suggesting that YR developed a more effective and coordinated antiviral response. Pathway enrichment in YD versus YR further substantiated this observation. By comparison, YC showed upregulation of DE PCGs in metabolic and synaptic processes, consistent with stable cellular homeostasis and efficient metabolic regulation in uninfected pigs. Moreover, YC displayed fewer upregulated lncRNAs than YD and YR, suggesting a more balanced state focused on metabolic and cellular communication. Collectively, these findings suggest that YD may rely heavily on inflammatory responses and cell apoptosis to counteract PEDV, and YR may mount a balanced and coordinated immune response. The YC group, on the other hand, may prioritize metabolic and synaptic processes to maintain cellular homeostasis and respond to the pathogen.

Transcriptomic comparisons between Yorkshire (YD, YR, and YC) and Chinese Min (MD, MR, and MC) piglets revealed significant differences in molecular expression patterns in response to PEDV, highlighting distinct strategies employed by the two breeds for immune response and disease resistance. These differences likely reflect their distinct genetic and physiological characteristics. In YC vs. MC, YC exhibited a gene expression profile suggestive of a proactive immune stance, with upregulated cytokine-cytokine receptor interactions pathways. However, this profile may represent a reactive rather than proactive approach to combating infections, potentially indicating predisposition to inflammation. In contrast, MC upregulated pathways related to metabolic processes and epithelial tight junctions, supporting stable cellular environment and effective barrier function against pathogens. The YD versus MD comparison revealed a significant number of downregulated PCGs. This implied a suppression of cellular functions, which might contribute to a compromised response to PEDV infection in Yorkshire pigs. Upregulated PCGs were involved in oxidative phosphorylation and pyrogenesis, reflecting cellular stress response and inflammatory states. In contrast, MD upregulated pathways such as ECM-receptor interaction and PI3K-Akt signaling, favoring intercellular communication and metabolic regulation. In YR versus MR, upregulated PCGs in YR were related to immune response pathways, consistent with active defense mechanism. However, these immune response pathways might imply a more inflammatory response that could be detrimental in the long term. MR, by contrast, showed upregulation of metabolic pathways, including metabolic pathways and glycerophospholipid metabolism, which were critical for cellular homeostasis and energy metabolism. This metabolic focus might provide the MR group with an advantage in sustaining vital cellular functions during PEDV infection (Wang and Green 2012; Buck et al. 2015; O’Neill et al. 2016; O’Neill and Pearce 2016; Muri and Kopf 2021). These findings emphasized that Yorkshire piglets rely heavily on immune activation and inflammation, which might be effective in combating infections but come at a cost of tissue damage and dysregulated metabolism. In contrast, Chinese Min piglets seemed to employ strategies centered on metabolic regulation and epithelial integrity, which contributed to a more stable and controlled response to PEDV infection, potentially minimizing tissue damage and enhancing recovery.

The *in vitro* interference model provided important insights into how the virus affected host cells. The intestinal transparency observed in infected pigs was consistent with the findings that PEDV suppressed IPEC-J2 proliferation and promoted apoptosis, indicating a relationship between cellular responses and the disease’s etiology. *G6PC3* deficiency causes severe congenital neutropenia (SCN), which can lead to inflammatory bowel disease (IBD) (Cullinane et al. 2011; Hayee et al. 2011; Smith et al. 2012; Bégin et al. 2013; Desplantes et al. 2014; Hudspeth et al. 2014; Kiykim et al. 2015; Glasser et al. 2016; Bolton et al. 2020). It is also associated with impaired bacterial clearance due to increased mTOR signaling (Bolton et al. 2022). In this study, *G6PC3* was implicated in multiple pathways, including the PI3K-Akt signaling pathway, AMPK signaling pathway, glycolysis/gluconeogenesis, insulin signaling pathway, galactose metabolism, and FoxO signaling pathway. Notably, *G6PC3* was significantly upregulated in both the death and resistance groups of Chinese Min piglets compared with Yorkshire piglets, suggesting an essential role in meeting the metabolic demands of immune cells during PEDV infection. Knockdown of *G6PC3* in IPEC-J2 reduced its expression without impairing cell viability, but markedly increased PEDV replication and N gene expression, confirming that G6PC3 restricts viral replication. In parallel, knockdown suppressed expression of key cytokines (*IL-1β, IL-6, IL-18*, and *IFN-β*), critical mediators of innate immunity. *IL-1β, IL-6,* and *IL-18* drive broad-spectrum inflammatory responses, while *IFN-α* and *IFN-β* form the first line of defense against viral pathogens (Bourgeois et al. 2011). Furthermore, *G6PC3* knockdown decreased expression of genes in the glycolysis/gluconeogenesis pathway (*ACSS1*, *ENO1*, *PFKL*, and *PKM),* suggesting that *G6PC3* might limit PEDV by modulating the glycolysis/gluconeogenesis pathway and cytokine production. The marked upregulation of *G6PC3* in Chinese Min piglets, which sustained less tissue damage upon PEDV infection, supported its role in enhancing resistance, likely through metabolic support of mucosal immunity. Transcriptomic analysis further identified *G6PC3* as a target gene of lncRNA *ENSSSCG00000055033*, and functional assays confirmed that this lncRNA positively regulates *G6PC3* expression. In summary, lncRNA *ENSSSCG00000055033* regulated *G6PC3* expression to enhance glycolysis/gluconeogenesis pathway and promote cytokine expression, thus inhibiting PEDV replication in IPEC-J2. Further research is warranted to elucidate the precise mechanisms by which *G6PC3* contributes to PEDV resistance.

## Conclusions

In conclusion, this study highlighted distinct transcriptional and immunological responses to PEDV infection in Yorkshire and Chinese Min piglets. Yorkshire piglets relied predominantly on T cell-mediated immunity and inflammatory pathways, which were associated with greater tissue damage. In contrast, Chinese Min piglets exhibited enhanced mucosal immunity and metabolic regulation, thereby conferring stronger resistance and preservation of tissue integrity. We identified *G6PC3* as a critical gene in PEDV resistance, regulated by the lncRNA *ENSSSCG00000055033*, through its role in glycolysis and immune response modulation. These findings provide novel insights into the genetic basis of PEDV resilience and contribute to understanding the breed-specific immune responses.

## Supplementary Material

Table S1 Sequence information of small interfering RNAs and primers.xlsx

## Data Availability

Data related to this article have been deposited in the Figshare repository and is available at https://doi.org/10.6084/m9.figshare.30208348. The dataset includes the identified protein-coding genes (PCGs) and long non-coding RNAs (lncRNAs) from Yorkshire piglets, the differentially expressed PCGs and lncRNAs characterized in this study, the predicted target genes of DE lncRNAs, and the results of GO/KEGG functional enrichment analysis. All raw RNA sequencing data generated in this study have been submitted to the National Center for Biotechnology Information Sequence Read Archive (NCBI SRA) database under BioProject numbers PRJNA842216 and PRJNA898078. The sequence data of the G2a strain are available from the corresponding author upon reasonable request.
